# Albumin infusion may decrease the incidence and severity of overt hepatic encephalopathy in liver cirrhosis

**DOI:** 10.18632/aging.102335

**Published:** 2019-10-08

**Authors:** Zhaohui Bai, Mauro Bernardi, Eric M. Yoshida, Hongyu Li, Xiaozhong Guo, Nahum Méndez-Sánchez, Yingying Li, Ran Wang, Jiao Deng, Xingshun Qi

**Affiliations:** 1 Department of Gastroenterology, General Hospital of Northern Theater Command (formerly called General Hospital of Shenyang Military Area), Shenyang, P.R. China; 2 Postgraduate College, Shenyang Pharmaceutical University, Shenyang, P.R. China; 3 Department of Medical and Surgical Sciences, University of Bologna, Bologna, Italy; 4 Division of Gastroenterology, Vancouver General Hospital, Vancouver, British Columbia, Canada; 5 Liver Research Unit, Medica Sur Clinic and Foundation and Faculty of Medicine, National Autonomous University of Mexico, Mexico; 6 Department of Pharmacology, General Hospital of Northern Theater Command, (formerly called General Hospital of Shenyang Military Area), Shenyang, P.R. China

**Keywords:** cirrhosis, hepatic encephalopathy, management, albumin

## Abstract

Background: The role of human albumin infusion for the prevention and treatment of overt hepatic encephalopathy (HE) in liver cirrhosis remains unclear. Results: Among the 708 patients without pre-existing overt HE, albumin infusion significantly decreased the incidence of overt HE (4.20% versus 12.70%, P<0.001) and in-hospital mortality (1.70% versus 5.40%, P=0.008). Among the 182 patients with overt HE at admission or during hospitalization, albumin infusion significantly improved overt HE (84.60% versus 68.10%, P=0.009) and decreased in-hospital mortality (7.70% versus 19.80%, P=0.018). Meta-analysis of 6 studies found that albumin infusion might decrease the risk of overt HE (OR=1.63, P=0.07), but the difference was not statistically significant. Meta-analysis of 3 studies found that albumin infusion significantly improved overt HE (OR=2.40, P=0.04). Conclusions: Based on the results of our retrospective study and meta-analysis, albumin infusion might prevent from the occurrence of overt HE and improve the severity of overt HE in cirrhosis. Our retrospective study also suggested that albumin infusion improved the outcomes of cirrhotic patients regardless of overt HE. Methods: Cirrhotic patients consecutively admitted between January 2010 and June 2014 were considered in a retrospective study. A 1:1 propensity score matching analysis was performed. Additionally, publications regarding albumin infusion for the management of overt HE were systematically searched. Meta-analyses were performed by random-effect model. Odds ratio (OR) was calculated.

## INTRODUCTION

Hepatic encephalopathy (HE) is a disorder of the brain caused by hepatic insufficiency and/or portosystemic shunting. Manifestations of HE include a wide range of neurological or psychiatric abnormalities, affecting motor, cognitive, and mental neuropsychiatric function [1]. Patients with covert HE have neuropsychological and/or neurophysiological abnormalities but without disorientation or asterixis. By contrast, patients with overt HE have obvious clinical signs of HE, which may present with directional and computational impairment, asterixis, drowsiness, and even coma. According to the underlying liver disease, HE can be divided into three types, including type A (acute liver failure), type B (portosystemic shunting), and type C (cirrhosis) [1]. The severity of overt HE is classified into grade I, II, III, and IV according to the West-Haven criteria [2]. The incidence of HE in patients with liver cirrhosis is reportedly 30-45% [3–5]. The one-year survival rate of patients with HE is less than 50%, and the three-year survival rate is less than 25% [6]. HE contributes a significant burden on healthcare systems. In 2014, the estimated national economic burden of hospitalizations with HE reached $11.9 billion in the USA [7].

Potential pathogeneses of HE mainly include hyperammonemia [8, 9], increased synthesis of gamma-aminobutyric acid (GABA) and pseudo neurotransmitters [10, 11], as well as consequences of bacterial infection and inflammatory processes [12, 13]. As for the excess systemic ammonia hypothesis, the drugs for management of HE mainly include lactulose [14, 15], rifaximin [16], and probiotics [17], all of which reduce ammonia production and absorption, as well as L-ornithine-L-aspartate (LOLA) [18] which increases ammonia clearance. Among them, lactulose and rifaximin are approved as the first-line choice of treatment for HE by the American Food and Drug Administration as well as other national drug regulatory agencies [19]. As for the GABA and pseudo neurotransmitter hypothesis, the most widely studied drug for management of HE is flumazenil that can block the GABA neural pathway, thereby improving the inhibition of central nervous system [20]. As for the bacterial infection and inflammatory process hypothesis, the substances that can eliminate inflammatory mediators and suppress oxidative stress should be considered. Recently, it has been reported that albumin can improve systemic inflammatory responses [21], which, in theory, could potentially be an effective therapy for the management of HE. However, the role of albumin infusion in the management of HE remains controversial. Among the studies regarding preventive role of albumin infusion, Sola et al. found that albumin could not prevent from HE in liver cirrhosis [22], but several studies suggested that long-term infusion of albumin could decrease the risk of HE in decompensated cirrhosis [23, 24]. Among the studies investigating the therapeutic role of albumin infusion, Simon-Talero et al. reported a poor therapeutic effect of albumin infusion on HE (but with a significant improvement in 90 day survival) [25], but several studies suggested that albumin infusion could improve both HE and survival in patients with decompensated liver cirrhosis [26, 27]. Currently, the use of albumin infusion has not been recommended for management of HE by the AASLD-EASL practice guideline [1]. By comparison, the Italian Association for the Study of the Liver (AISF) suggested that albumin could decrease the incidence of grade III and IV type C overt HE in cirrhotic patients with ascites [28].

Our work included a retrospective observational study and a systematic review with meta-analysis to clarify the role of albumin infusion in prevention and treatment of overt HE in liver cirrhosis.

## RESULTS

### Observational study

### Patient selection

**Figure 1:**
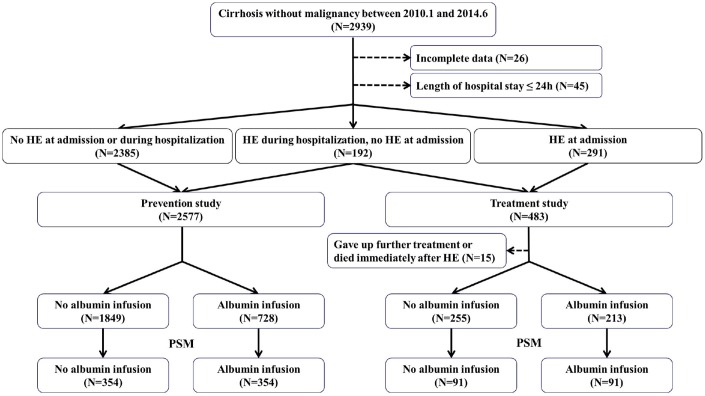
**Flow chart of patient selection in our observational study.**

Between January 2010 and June 2014, a total of 2868 cirrhotic patients were included (Figure 1). In the prevention study, 2577 cirrhotic patients without overt HE at admission were included. There were 728 patients in the albumin infusion group and 1849 patients in the control group. In the treatment study, 468 cirrhotic patients with overt HE at or after admission were included. There were 213 patients in the albumin infusion group and 255 patients in the control group.

### Prevention study

After a 1:1 propensity score matching (PSM) analysis, a total of 708 patients were included. Three hundred and fifty-four patients were matched in each group (Figure 1). Baseline characteristics were described in Table 1. Median total dosage of albumin was 30g (range: 5–210g) in the albumin infusion group. Median Child-Pugh score was 8. Median MELD score was 7.30. The incidence of overt HE and the in-hospital mortality were 8.50% (60/708) and 3.50% (25/708), respectively (Table 2).

**Table 1:** Prevention study – Baseline characteristics in albumin and control groups after PSM.

**Variables**	**No. Pts**	**Overall**	**No. Pts**	**Albumin group**	**No. Pts**	**Control group**	**P value**
**Age (years)**	708	56.80 (14.37-89.19) 57.82±12.54	354	57.63 (17.41-87.13) 58.12±12.57	354	56.45 (14.37-89.19) 57.52±12.52	0.454
**Sex (male) (%)**	708	440 (62.10%)	354	226 (63.80%)	354	214 (60.50%)	0.352
**Etiology of Liver Diseases**	708		354		354		0.207
HBV (%)		192 (27.10%)		102 (28.80%)		90 (25.40%)	
HCV (%)		43 (6.10%)		22 (6.20%)		21 (5.90%)	
Alcohol Abuse (%)		150 (21.20%)		63 (17.80%)		87 (24.60%)	
HBV+Alcohol Abuse (%)		68 (9.60%)		42 (11.90%)		26 (7.30%)	
HCV+Alcohol Abuse (%)		11 (1.60%)		5 (1.40%)		6 (1.70%)	
Drug Related Liver Diseases (%)		9 (1.30%)		6 (1.70%)		3 (0.80%)	
Autoimmune Liver Diseases (%)		46 (6.50%)		22 (6.20%)		24 (6.80%)	
Other or Unknown Etiology (%)		189 (26.70%)		92 (26.00%)		97 (27.40%)	
**Potential inducement of HE**							
AUGIB (%)	708	196 (27.70%)	354	88 (24.90%)	354	108 (30.50%)	0.093
Infection (%)	708	121 (17.10%)	354	58 (16.40%)	354	63 (17.80%)	0.618
Ascites (%)	708	454 (64.10%)	354	231 (65.30%)	354	223 (63.00%)	0.531
Ascites (None/Mild/ Moderate+Severe) (%)	708	254 (35.90%)/90 (12.70%)/364 (51.40%)	354	123 (34.80%)/43 (12.10%)/188 (53.10%)	354	131 (37.00%)/47 (13.30%)/176 (49.70%)	0.662
Abdominal Paracentesis (%)	708	73 (10.30%)	354	45 (12.70%)	354	28 (7.90%)	*0.036*
**Laboratory Tests**							
RBC (10^12^/L)	706	2.98 (1.01-5.89) 3.00±0.78	353	3.05 (1.25-5.57) 3.05±0.73	353	2.87 (1.01-5.89) 2.94±0.83	*0.024*
Hb (g/L)	706	92.00 (27.00-176.00) 91.85±28.07	353	95.00 (29.00-169.00) 94.42±26.89	353	87.00 (27.00-176.00) 89.29±29.02	*0.011*
WBC (10^9^/L)	706	4.10 (0.80-33.00) 5.16±3.73	353	3.80 (0.80-26.00) 4.88±3.49	353	4.30 (1.00-33.00) 5.44±3.95	*0.030*
PLT (10^9^/L)	706	74.00 (3.00-467.00) 93.24±68.27	353	73.00 (17.00-394.00) 87.61±57.35	353	76.00 (3.00-467.00) 98.86±77.35	0.183
TBIL (μmol/L)	708	23.30 (1.90-809.80) 44.20±70.97	354	24.30 (2.00-379.50) 38.44±47.17	354	22.40 (1.90-809.80) 49.96±88.30	0.737
DBIL (μmol/L)	706	10.40 (0.30-562.80) 24.37±49.21	354	10.80 (0.30-276.20) 20.51±33.10	352	10.00 (0.40-562.80) 28.24±61.08	0.772
IBIL (μmol/L)	706	13.05 (0.90-265.30) 19.91±25.55	354	13.35 (0.90-124.70) 17.93±16.99	352	12.45 (1.30-265.30) 21.90±31.85	0.801
ALB (g/L)	708	30.15 (0.40-50.00) 30.20±6.28	354	29.70 (13.10-50.00) 30.06±6.42	354	30.60 (0.40-45.50) 30.34±6.14	0.191
ALT (U/L)	708	25.00 (4.00-1460.00) 41.57±84.90	354	26.00 (4.00-495.00) 37.45±44.36	354	25.00 (5.00-1460.00) 45.69±111.51	0.985
AST (U/L)	708	37.00 (7.00-1366.00) 58.32±80.67	354	37.00 (7.00-649.00) 56.51±58.18	354	36.00 (8.00-1366.00) 60.11±98.20	0.202
AKP (U/L)	708	86.00 (17.00-969.00) 124.03±114.25	354	84.75 (26.00-739.70) 122.86±113.31	354	89.00 (17.00-969.00) 125.20±115.33	0.531
GGT(U/L)	708	51.00 (6.00-2280.00) 119.25±193.61	354	48.00 (6.00-1221.00) 109.05±174.79	354	58.00 (8.00-2280.00) 129.45±210.51	0.117
BUN (mmol/L)	708	6.08 (1.72-62.45) 7.80±6.45	354	6.18 (1.72-44.34) 7.69±5.81	354	6.00 (1.81-62.45) 7.91±7.03	0.307
Scr (μmol/L)	708	61.00 (23.00-933.90) 89.25±112.89	354	61.00 (27.40-702.00) 83.34±86.44	354	60.95 (23.00-933.90) 95.15±134.10	0.430
K (mmol/L)	701	3.99 (2.13-8.28) 4.00±0.58	353	4.00 (2.68-8.28) 4.01±0.54	348	3.99 (2.13-7.24) 3.99±0.62	0.655
Na (mmol/L)	701	138.70 (118.90-157.80) 138.07±4.45	353	138.40 (124.60-157.80) 138.14±4.33	348	138.95 (118.90-152.40) 138.01±4.56	0.737
Ammonia (μmol/L)	343	36.00 (9.00-791.60) 47.27±58.18	164	35.00 (9.00-791.60) 45.79±71.75	179	36.00 (9.00-238.00) 48.62±42.26	0.228
PT (seconds)	708	15.80 (10.50-42.50) 16.45±3.49	354	15.80 (11.00-33.70) 16.30±3.05	354	15.80 (10.50-42.50) 16.59±3.89	0.850
INR	708	1.27 (0.76-4.75) 1.35±0.39	354	1.27 (0.79-3.28) 1.33±0.34	354	1.26 (0.76-4.75) 1.37±0.44	0.769
APTT (seconds)	708	42.60 (27.30-472.40) 44.73±19.51	354	43.45 (28.80-83.30) 43.98±7.34	354	41.90 (27.30-472.40) 45.49±26.60	0.050
**Child-Pugh Score**	708	8 (5-13) 7.96±1.71	354	8 (5-13) 7.97±1.69	354	8 (5-13) 7.96±1.74	0.677
**Child-Pugh Class A/B/C (%)**	708	143 (20.20%)/444 (62.70%)/121 (17.10%)	354	73 (20.60%)/224 (63.30%)/57 (16.10%)	354	70 (19.80%)/220 (62.10%)/64 (18.10%)	0.777
**MELD Score**	708	7.30 (-5.64-38.80) 8.31±6.76	354	7.19 (-5.64-26.71) 8.10±6.03	354	7.33 (-5.08-38.80) 8.51±7.41	0.986

Abbreviations: Pts: Patients; HBV: Hepatic B Virus; HCV: Hepatic C Virus; AUGIB: Acute Upper Gastrointestinal Bleeding, RBC: Red Blood Cell; Hb: Hemoglobin; WBC: White Blood Cell; PLT: Platelet Count; TBIL: Total Bilirubin; DBIL: Direct Bilirubin; IBIL: Indirect Bilirubin; ALB: Albumin; ALT: Alanine Aminotransferase; AST: Aspartate Aminotransferase; AKP: Alkaline Phosphatase; GGT: Gamma-Glutamyl Transpeptidase; BUN: Blood Urea Nitrogen; Scr: Serum Creatinine; K: Potassium; Na: Sodium; PT, Prothrombin Time; INR: International Standardization Ratio; APTT: Activated Partial Thromboplastin Time; MELD: Model for End-stage Liver Disease.

**Table 2:** Prevention study - Interventions and outcomes of patients during hospitalizations in albumin and control groups after PSM.

**Variables**	**No. Pts**	**Overall**	**No. Pts**	**Albumin group**	**No. Pts**	**Control group**	**P value**
Frozen Plasma Infusion (%)	708	136 (19.20%)	354	61 (17.20%)	354	75 (21.20%)	0.182
Lactulose (%)	708	128 (18.10%)	354	61 (17.20%)	354	67 (18.90%)	0.558
L-ornithine-L-aspartate (%)	708	104 (14.70%)	354	48 (13.60%)	354	56 (15.80%)	0.396
Compound Amino Acid (6AA) (%)	708	95 (13.40%)	354	44 (12.40%)	354	51 (14.40%)	0.440
Compound Amino Acid (15HBC) (%)	708	268 (37.90%)	354	126 (35.60%)	354	142 (40.10%)	0.215
Compound Amino Acid (18AA) (%)	708	98 (13.80%)	354	42 (11.90%)	354	56 (15.80%)	0.128
Arginine (%)	708	73 (10.30%)	354	32 (9.00%)	354	41 (11.60%)	0.266
Alanylglutamine (%)	708	121 (17.10%)	354	55 (15.50%)	354	66 (18.60%)	0.272
Antibiotic (%)	708	376 (53.10%)	354	175 (49.40%)	354	201 (56.80%)	0.050
Dosage of Albumin Infused (g)	354	30 (5-210) 42.75±35.47	NA	30 (5-210) 42.75±35.47	354	NA	NA
Incidence of Overt HE (%)	708	60 (8.50%)	354	15 (4.20%)	354	45 (12.70%)	*<0.001*
Time from Admission to HE (days)	60	4.21 (0.24-84.79) 8.54±13.09	15	5.61 (1.62-47.99) 10.52±11.73	45	3.73 (0.24-84.79) 7.89±13.57	0.066
In-hospital Death (%)	708	25 (3.50%)	354	6 (1.70%)	354	19 (5.40%)	*0.008*

Abbreviations: NA: Not Applicable; HE: Hepatic Encephalopathy.

The albumin infusion group had significantly higher red blood cell (P=0.024) and hemoglobin (P=0.011) and lower white blood cell (P=0.030) than the control group (Table 1). The albumin infusion group had a significantly higher proportion of abdominal paracentesis than the control group (12.70% versus 7.90%, P=0.036) (Table 2). The albumin infusion group had significantly lower incidence of overt HE (4.20% versus 12.70%, P<0.001) and in-hospital mortality (1.70% versus 5.40%, P=0.008) than the control group (Table 2).

### Treatment study

After a 1:1 PSM analysis, a total of 182 patients were included. Ninety-one patients were matched in each group (Figure 1). Baseline characteristics were described in Table 3. Median total dosage of albumin was 40g (range: 10–250g) in the albumin infusion group. Median Child-Pugh score was 10. Median model for end-stage liver disease (MELD) score was 12.21. The rate of HE improvement and the in-hospital mortality were 76.40% (139/182) and 13.70% (25/182), respectively (Table 4).

**Table 3:** Treatment study - Baseline characteristics in albumin and control groups after PSM.

**Variables**	**No. Pts**	**Overall**	**No. Pts**	**Albumin group**	**No. Pts**	**Control group**	**P value**
**Age (years)**	182	57.04 (30.78-84.77) 58.93±11.81	91	56.01 (38.06-84.74) 58.99±11.83	91	58.17 (30.78-84.77) 58.86±11.85	0.927
**Sex (male) (%)**	182	123 (67.60%)	91	59 (64.80%)	91	64 (70.30%)	0.428
**Etiology of Liver Diseases**	182		91		91		0.069
HBV (%)		44 (24.20%)		28 (30.80%)		16 (17.60%)	
HCV (%)		10 (5.50%)		5 (5.50%)		5 (5.50%)	
Alcohol Abuse (%)		49 (26.90%)		18 (19.80%)		31 (34.10%)	
HBV+Alcohol Abuse (%)		22 (12.10%)		12 (13.20%)		10 (11.00%)	
HCV+Alcohol Abuse (%)		2 (1.10%)		1 (1.10%)		1 (1.10%)	
Drug Related Liver Diseases (%)		2 (1.10%)		0 (0.00%)		2 (2.20%)	
Autoimmune Liver Diseases (%)		15 (8.20%)		11 (12.10%)		4 (4.40%)	
Other or Unknown Etiology (%)		38 (20.90%)		16 (17.60%)		22 (24.20%)	
**Potential inducement of HE**							
AUGIB (%)	182	58 (31.90%)	91	29 (31.90%)	91	29 (31.90%)	1.000
Infection (%)	182	47 (25.80%)	91	21 (23.10%)	91	26 (28.60%)	0.397
Ascites (%)	182	136 (74.70%)	91	67 (73.60%)	91	69 (75.80%)	0.733
Ascites (None/Mild/ Moderate+Severe) (%)	182	46 (25.30%)/22 (12.10%)/114 (62.60%)	91	24 (26.40%)/10 (11.00%)/57 (62.60%)	91	22 (24.20%)/12 (13.20%)/57 (62.60%)	0.874
Abdominal Paracentesis (%)	182	28 (15.40%)	91	15 (16.50%)	91	13 (14.30%)	0.681
**Overt HE**	182		91		91		1.000
Grade 1-2 (%)		124 (68.10%)		62 (68.10%)		62 (68.10%)	
Grade 3-4 (%)		58 (31.90%)		29 (31.90%)		29 (31.90%)	
**Laboratory Tests**							
RBC (10^12^/L)	181	2.69 (1.19-5.33) 2.76±0.72	90	2.61 (1.19-4.54) 2.70±0.72	91	2.80 (1.52-5.33) 2.82±0.73	0.241
Hb (g/L)	181	89.00 (42.00-157.00) 89.84±24.91	90	89.50 (42.00-144.00) 87.72±22.93	91	87.00 (43.00-157.00) 91.93±26.69	0.367
WBC (10^9^/L)	181	4.60 (1.10-31.40) 5.90±4.23	90	4.40 (1.10-31.40) 5.64±4.75	91	5.00 (1.30-21.00) 6.16±3.67	0.081
PLT (10^9^/L)	181	76.00 (13.00-314.00) 82.55±49.55	90	68.50 (13.00-282.00) 77.98±51.20	91	80.00 (17.00-314.00) 87.08±47.71	0.077
TBIL (μmol/L)	182	38.95 (5.90-607.80) 62.40±76.80	91	37.10 (5.90-607.80) 61.37±85.35	91	39.30 (7.70-383.20) 63.43±67.64	0.437
DBIL (μmol/L)	182	17.70 (0.70-331.70) 33.86±48.78	91	16.80 (0.70-331.70) 32.02±52.69	91	18.80 (3.60-242.50) 35.71±44.75	0.297
IBIL (μmol/L)	182	19.10 (1.90-276.10) 28.54±31.10	91	18.50 (1.90-276.10) 29.36±35.71	91	19.70 (3.30-140.70) 27.72±25.84	0.842
ALB (g/L)	182	27.10 (0.40-42.50) 26.79±6.05	91	26.50 (10.50-42.50) 26.55±6.13	91	27.30 (0.40-42.40) 27.03±5.99	0.465
ALT (U/L)	182	26.00 (8.00-748.00) 41.63±70.97	91	25.00 (8.00-201.00) 35.88±33.10	91	27.00 (9.00-748.00) 47.38±74.70	0.467
AST (U/L)	182	43.50 (11.00-1230.00) 66.79±108.64	91	42.00 (14.00-228.00) 54.42±40.39	91	44.00 (11.00-1230.00) 79.15±147.63	0.563
AKP (U/L)	182	91.00 (17.00-470.00) 111.22±68.39	91	88.00 (25.00-402.00) 108.22±66.64	91	98.00 (17.00-470.00) 114.23±70.34	0.462
GGT (U/L)	182	43.00 (8.00-1102.00) 84.43±124.90	91	41.00 (9.00-702.00) 79.67±107.84	91	46.00 (8.00-1102.00) 89.19±140.36	0.402
BUN (mmol/L)	182	7.93 (1.58-62.45) 10.83±9.35	91	7.94 (1.72-46.02) 10.15±8.04	91	7.79 (1.58-62.45) 11.50±10.50	0.847
Scr (μmol/L)	182	69.50 (24.00-1069.00) 103.57±119.91	91	71.00 (25.00-533.60) 93.35±77.32	91	68.00 (24.00-1069.00) 113.79±150.75	0.830
K (mmol/L)	182	4.10 (2.25-5.99) 4.15±0.67	91	4.10 (2.65-5.57) 4.17±0.59	91	4.10 (2.25-5.99) 4.12±0.75	0.694
Na (mmol/L)	182	137.70 (83.00-152.40) 136.42±6.63	91	138.00 (83.00-146.60) 136.60±7.47	91	137.10 (121.00-152.40) 136.23±5.71	0.287
Ammonia (μmol/L)	177	73.00 (5.30-415.00) 81.47±57.54	88	75.50 (5.30-325.00) 80.87±58.85	89	72.00 (9.00-415.00) 82.07±56.54	0.818
PT (seconds)	182	18.20 (11.50-62.80) 19.50±6.11	91	18.30 (11.50-62.80) 19.87±7.36	91	18.00 (12.30-36.30) 19.14±4.55	0.821
INR	182	1.55 (0.88-7.96) 1.70±0.80	91	1.55 (0.88-7.96) 1.75±0.97	91	1.52 (0.94-3.60) 1.66±0.52	0.954
APTT (seconds)	182	45.95 (29.30-180.00) 48.98±14.89	91	47.00 (29.30-180.00) 49.75±17.18	91	44.80 (30.00-114.90) 48.22±12.22	0.495
**Child-Pugh Score**	182	10 (5-14) 9.95±2.01	91	10 (5-14) 9.92±1.90	91	10 (6-14) 9.98±2.13	0.895
**Child-Pugh Class A/B/C (%)**	182	4 (2.20%)/73 (40.10%)/105 (57.70%)	91	2 (2.20%)/36 (39.60%)/53 (58.20%)	91	2 (2.20%)/37 (40.70%)/52 (57.10%)	0.988
**MELD Score**	182	12.21 (0.13-42.68) 13.50±8.40	91	11.92 (0.79-42.68) 13.07±7.96	91	12.50 (0.13-38.80) 13.94±8.84	0.676

Abbreviations: Pts: Patients; HBV: Hepatic B Virus; HCV: Hepatic C Virus; AUGIB: Acute Upper Gastrointestinal Bleeding; RBC: Red Blood Cell; Hb: Hemoglobin; WBC: White Blood Cell; PLT: Platelet Count; TBIL: Total Bilirubin; DBIL: Direct Bilirubin; IBIL: Indirect Bilirubin; ALB: Albumin; ALT: Alanine Aminotransferase; AST: Aspartate Aminotransferase; AKP: Alkaline Phosphatase; GGT: Gamma-Glutamyl Transpeptidase; BUN: Blood Urea Nitrogen; Scr: Serum Creatinine; K: Potassium; Na: Sodium; PT, Prothrombin Time; INR: International Standardization Ratio; APTT: Activated Partial Thromboplastin Time; MELD: Model for End-stage Liver Disease.

**Table 4:** Treatment study- Interventions and outcomes of patients during hospitalizations in albumin and control groups after PSM.

**Variables**	**No. Pts**	**Overall**	**No. Pts**	**Albumin group**	**No. Pts**	**Control group**	**P value**
Frozen Plasma Infusion (%)	182	54 (29.70%)	91	29 (31.90%)	91	25 (27.50%)	0.516
Lactulose (%)	182	87 (47.80%)	91	45 (45.50%)	91	42 (46.20%)	0.656
L-ornithine-L-aspartate (%)	182	132 (72.50%)	91	69 (75.80%)	91	63 (69.20%)	0.319
Compound Amino Acid (6AA) (%)	182	115 (63.20%)	91	56 (61.50%)	91	59 (64.80%)	0.645
Compound Amino Acid (15HBC) (%)	182	41 (22.50%)	91	19 (20.90%)	91	22 (24.20%)	0.595
Compound Amino Acid (18AA) (%)	182	14 (7.70%)	91	6 (6.60%)	91	8 (8.80%)	0.578
Arginine (%)	182	105 (57.70%)	91	51 (56.00%)	91	54 (59.30%)	0.653
Alanylglutamine (%)	182	20 (11.00%)	91	12 (13.20%)	91	8 (8.80%)	0.343
Antibiotics (%)	182	101 (55.50%)	91	48 (52.70%)	91	53 (58.20%)	0.456
Dosage of Albumin Infused (g)	91	40 (10-250) 46.48±37.16	NA	40 (10-250) 46.48±37.16	91	NA	NA
Improvement of Overt HE (%)	182	139 (76.40%)	91	77 (84.60%)	91	62 (68.10%)	*0.009*
In-hospital Death (%)	182	25 (13.70%)	91	7 (7.70%)	91	18 (19.80%)	*0.018*

Abbreviations: NA: Not Applicable; HE: Hepatic Encephalopathy.

No significant difference in the baseline characteristics and interventions was observed between albumin infusion and control groups (P>0.05, in all comparisons) (Table 3 and Table 4). The albumin infusion group had a significantly higher rate of overt HE improvement (84.60% versus 68.10%, P=0.009) and a significantly lower in-hospital mortality (7.70% versus 19.80%, P=0.018) than the control group (Table 4).

### Systematic review with meta-analysis

### Study selection

A total of 3496 studies were identified in EMBASE, PubMed, and Cochrane Library databases. Finally, 9 studies were eligible, including 6 studies investigating the prevention of HE and 3 studies investigating the treatment of HE (Supplementary Figure 1). Characteristics of the studies were summarized in Table 5. Among all of these included studies, the severity of HE was assessed based on the West-Haven criteria [2]. Inclusion and exclusion criteria were summarized in Supplementary Table 1. Characteristics of patients were summarized in Supplementary Table 2. Biochemical variables for treatment studies were summarized in Supplementary Table 3.

**Table 5:** Characteristics of studies.

**First Author (year)**	**Country**	**Study Design**	**Enrollment Period**	**Number of Patients**	**Dosage of Albumin Infused**	**Outcomes**	**Main Findings**
***Treatment study***							
Jalan (2004)	UK	Cohort	NA	15	*Albumin group*: to administer 4.5% albumin intravenously until the central venous pressure was sustained at 7–10mmHg. *Control group*: none.	Improvement of HE during 3 days.	Severity of HE was significantly improved in the albumin group at both 24 and 72h (P<0.01), which was not observed in the control group (P=0.21).
Simon-Talero (2013)	Spain	RCT	2009- 2012	56	*Albumin group*: day 1: albumin 1.5g/kg; day 3: albumin 1.0g/kg. *Control group*: none.	Presence of HE at day 4.	No difference in the proportion of patients without HE at day 4 between albumin and control groups (65.2% vs 57.1%, P=0.6).
Sharma (2017)	India	RCT	2015- 2016	120	*Albumin group*: albumin 1.5g/kg/day. *Control group*: none.	Recovery of HE during 10 days.	Difference in the rate of complete reversal of HE within 10 days was significant between albumin and control groups (75% vs 53.3%, P=0.03).
***Prevention study***							
Planas (1990)	Spain	RCT	NA	88	*Albumin group*: when 1L of ascites was removed, 8g albumin was infused. *Control group*: none.	Improvement of ascites. Incidence of HE.	Three patients developed HE during hospitalization in each group (albumin and control group).
Riggio (2015)	Italy	Cohort	NA	68	*Albumin group*: day 1, 2: albumin 1.0g/kg/day; day 4, 7, 14, 21, 28: albumin 0.5g/kg/day. *Control group*: none.	Incidence of overt HE during first month.	No difference in the incidence of HE between albumin and control groups during the first month (34% vs 31%) or during the whole follow-up (39% vs 48%).
Arora (2018)	India	RCT	NA	59	*Albumin group*: when 1L of ascites was removed, 8g albumin was infused. *Control group*: none.	Improvement of ascites. Incidence of HE.	No difference in the incidence of HE between albumin and control groups (6.7% vs 24%, P=0.06).
Caraceni (2018)	Italy	RCT	2011- 2015	431	*Albumin group*: albumin 40g twice per week for two weeks, and then 40g albumin per week. *Control group*: no additional use of albumin, except for standard medical treatment.	Improvement of ascites. Incidence of HE.	Difference in the incidence of HE was significant between albumin and control groups (rate ratio=0.48, 95%CI=0.37 to 0.63, P<0.001).
Sola (2018)	Spain	RCT	2008- 2015	173	*Albumin group*: albumin 40g per 15 days. *Control group*: none.	Incidence of complications of cirrhosis. Incidence of HE.	No difference in the incidence of HE between albumin and control groups (28% vs 24%, P=0.635).
Di Pascoli (2019)	Italy	Cohort	2012- 2016	70	*Albumin group*: albumin 20g twice per week; when 1L of ascites was removed and 6-8g albumin was infused. *Control group:* no additional use of albumin, except for standard medical treatment.	24-month mortality. Incidence of HE.	Difference in the incidence of HE was significant between albumin and control groups (26.9% vs 64.5%, P=0.016).

Abbreviations: HE: Hepatic Encephalopathy; RCT: Randomized Controlled Trial; NA: Not Available.

### Prevention study

A total of 889 patients were included. The sample size ranged from 59 to 431 among studies. Four studies were randomized controlled trials (RCTs), and 2 studies were cohort studies. All studies were published between 1990 and 2019.

In terms of random sequence generation and blinding of participants and personnel, 3 RCTs had a low risk of bias; as for allocation concealment, 2 RCTs had a low risk of bias; as for blinding of outcome assessment, incomplete outcome data, and selective reporting, all RCTs had a low risk of bias; in terms of other bias, all RCTs had an unclear risk of bias (Supplementary Figure 2A). The Riggio’s [29] and Di Pascoli’s [24] study scored 8 and 7 points according to the Newcastle-Ottawa Scale, respectively.

**Figure 2:**
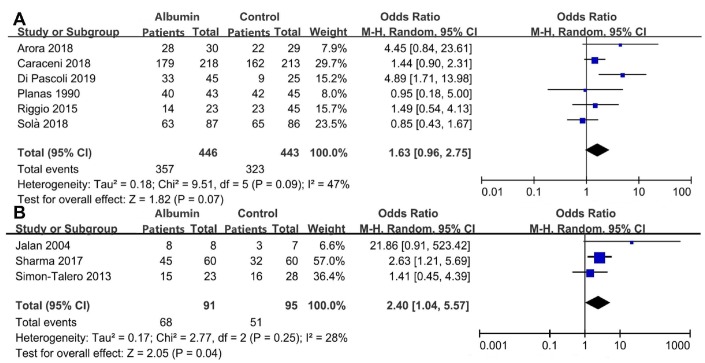
Meta-analyses regarding the prevention (**A**) and treatment (**B**) of overt HE.

The data regarding the development of overt HE was extracted in 6 studies including 889 patients [22–24, 29–31] (Supplementary Table 4). Albumin infusion might decrease the risk of overt HE in cirrhosis (OR=1.63, 95%CI=0.96 to 2.75, P=0.07), but the difference did not reach statistical significance (Figure 2A). There was a significant heterogeneity among studies (I^2^=47%, P=0.09). There was no significant publication bias (P=0.437) (Supplementary Figure 3).

### Treatment study

A total of 191 patients were included. The sample size ranged from 15 to 120 among studies. Two studies were RCTs, and 1 study was a cohort study. All studies were published between 2004 and 2017.

In terms of random sequence generation, allocation concealment, and blinding of participants and personnel, 1 RCT had a low risk of bias; in terms of blinding of outcome assessment, incomplete outcome data, and selective reporting, all RCTs had a low risk of bias; as for other bias, all RCTs studies had an unclear risk of bias (Supplementary Figure 2B). The Jalan’s [27] study scored 5 points according to the Newcastle-Ottawa Scale.

The data regarding the improvement of overt HE was explored in 3 studies including 191 patients [25–27] (Supplementary Table 5). Albumin infusion might increase the improvement of overt HE (odds ratio [OR]=2.40, 95% confidence interval [CI]=1.04 to 5.57, P=0.04) (Figure 2B). There was no significant heterogeneity (I^2^=28%, P=0.25). There was no significant publication bias (P=0.623) (Supplementary Figure 4).

Plasma ammonia level was explored in 3 studies including 191 patients [25–27]. Albumin infusion did not significantly change the ammonia level (mean difference [MD]=0.28, 95%CI=-3.03 to 3.58, P=0.87) (Supplementary Figure 5A). There was no significant heterogeneity (I^2^=0%, P=0.86).

Interleukin (IL)-6 level was explored in 2 studies including 176 patients [25, 26]. Albumin infusion significantly decreased the IL-6 level (MD=-6.20, 95%CI=-8.66 to -3.75, P<0.00001) (Supplementary Figure 5B). There was no significant heterogeneity (I^2^=0%, P=0.77).

tumor necrosis factor (TNF)-α level was explored in 2 studies including 176 patients [25, 26]. Albumin infusion might decrease the TNF-α level (MD=-1.08, 95%CI=-22.94 to 20.77, P=0.92) (Supplementary Figure 5C), but the difference was not statistically significant. There was a significant heterogeneity (I^2^=64%, P=0.10).

## DISCUSSION

Based on a single-center retrospective study involving 2868 cirrhotic patients and a meta-analysis of 9 studies, the main findings were as follows. First, albumin infusion was associated with reduced incidence and improvement of overt HE. Based on the results of our meta-analysis, the number needed to treat was 14 (95%CI=8 to 46) and 5 (95%CI=4 to 6) for preventing the development of overt HE and improving the severity of overt HE, respectively. Second, albumin infusion might be associated with reduced in-hospital mortality in cirrhotic patients with or without overt HE.

Our study had several features. First, our findings were not only derived from a relatively large number of original data obtained at our hospital, but also further confirmed by meta-analyses. Second, a 1:1 PSM analysis was employed to adjust the factors that might influence the outcomes of overt HE, including severity of liver dysfunction, infection, paracentesis, gastrointestinal bleeding, and drugs for HE. Third, our original study focused on the in-hospital outcomes. Fourth, our study objectives included both prevention and treatment of overt HE.

Albumin, which not only expands intravascular volume and improves microcirculation but also binds numerous substances, such as bile acids, nitric oxide, and cytokines [32, 33], has been widely employed for management of cirrhosis and portal hypertension related complications in real-world clinical practice. An US-based AASLD survey on the use of albumin in cirrhosis suggested that albumin was mainly employed for preventing post-paracentesis circulatory dysfunction (PPCD) and hepatorenal syndrome (HRS) and treating spontaneous bacterial peritonitis (SBP), HRS, hypotension, refractory ascites, hyponatremia, edema, and hypoalbuminemia [34]. Notably, only a few physicians prescribed albumin for HE. A European survey on use of albumin in cirrhosis suggested that albumin was mainly employed for preventing PPCD and renal failure and treating type-1 HRS, ascites, non-SBP bacterial infections, severe hyponatremia (<125mmol/L), HE, and hypoalbuminemia [35]. Among them, only a few indications have been recommended by the evidence-based guidelines. For example, the AASLD guideline recommends that albumin should be employed for large-volume paracentesis and HRS [36]; similarly, the EASL guideline suggests that albumin should be employed for the prevention of PPCD, SBP, and HRS [37]. By comparison, other indications, such as HE, hyponatremia, hypoalbuminemia, edema, and non-SBP bacterial infections, were not sufficiently supported by the currently available evidence. In our study, human albumin was prescribed at the discretion of attending physicians, and the primary indications for the use of albumin infusion mainly included post-paracentesis, ascites, and hypoalbuminemia.

Inflammatory markers and serum endotoxin levels are significantly increased in decompensated cirrhotic patients [21, 38, 39]. These cytokines may affect the integrity of the blood-brain barrier, and allow a large number of plasma ammonia ions enter into the brain, thereby causing HE [39, 40]. Recently, it has been reported that anti-TNF-α treatment can improve cognitive impairment in rats caused by chronic hyperammonemia-induced peripheral and central nervous system inflammation that can lead to neurotransmission and cognitive impairment [41]. Albumin is a multi-domain protein stabilized by 17 disulfide bonds where 34 cysteine residues are involved [42]. Among them, a free cysteine (Cys-34) can regulate inflammation [43] by reversibly binding to many inflammation mediators and transporting them to specific tissues or organs [44]. Supplementation of albumin in patients with cirrhosis can bind to inflammatory cytokines and acts to protect the blood-brain barrier [21, 45]. Our meta-analysis also suggested that albumin infusion might decrease IL-6 level.

Oxidative stress may also have a potential role in the pathogenesis of HE [46–49]. Albumin has a powerful antioxidant capacity [50]. Such an effect can be achieved by the abilities of albumin in binding to and inactivating free metals, such as copper and iron, which can catalyze the formation of reactive oxygen [51, 52], and capturing free radicals, such as reactive oxygen and nitrogen [53–55], which can damage astrocytes in patients with HE [48, 49].

Serum sodium abnormalities may be a risk factor for HE in cirrhosis [56, 57]. In Caraceni’s study, albumin infusion significantly reduced the incidence of hyponatremia [23]. And, a recent large-scale study also found that albumin infusion could improve hyponatremia in patients with cirrhosis [58].

It has been proposed that the effect of albumin infusion for hyponatremia in cirrhosis may be attributed to the correction of an impaired Gibbs-Donnan equilibrium that results in the imbalance of charged particles near the semipermeable membrane can alter fluid dynamics [59]. At the Gibbs-Donnan equilibrium, the chemical gradient is equal in magnitude and opposite in direction to the electrical gradient as described by the following equation: FE_m_=-RT ln([Na^+^]_1_ ⁄[Na^+^]_2_), where F is Faraday’s constant; R is ideal gas constant; T is absolute temperature; [Na^+^]_1_ is the sodium concentration in the protein-containing compartment; and [Na^+^]_2_ is the sodium concentration in the non-protein-containing compartment [59].

Major limitations of our study are as follows. In our retrospective study, the potential for patient selection bias and misclassification were unavoidable; additionally, the standard dosage of albumin was lacking, but the dosage was often dependent upon the physicians’ considerations. In our meta-analysis, a relatively small number of studies were included, and the patient characteristics, duration and dosage of albumin, and use of other drugs for management of overt HE were heterogeneous among them. Additionally, we have to acknowledge that the absolute number of patients who developed HE events, but not the cumulative incidence of HE or number of HE events, was extracted from the original papers. This is because the data expression is often heterogeneous among studies. For example, in the ANSWER study, the data regarding the incidence of complications were reported by “per person/year”. It should also be considered that this study only reported the occurrence of grade III or IV HE and the duration of the follow-up of patients who only received standard medical treatment was significantly shorter with respect to those who also received albumin. This may have influenced the results of the present meta-analysis, as the incidence of HE episodes assessed by Kaplan-Meier estimation showed a significantly lower value in the group who received human albumin. Therefore, the role of albumin administration in the prevention of HE warrants investigation in future studies.

In conclusion, albumin infusion may be effective for preventing the development of overt HE and improving the severity of overt HE in patients with cirrhosis. In future studies, the hypothesis should be confirmed that the benefit of albumin in management of HE may be achieved by regulating inflammation and anti-oxidative stress and improving sodium concentration (Supplementary Figure 6). Considering a relatively high cost of human albumin, especially the need of a large dosage of albumin infused, the cost-effectiveness of albumin infusion in such patients should be further explored in the future. Additionally, albumin infusion may also decrease the in-hospital mortality of patients with cirrhosis regardless of overt HE. More prospective studies are needed to explore the role of albumin infusion for management of HE in cirrhotic patients.

## MATERIALS AND METHODS

### Observational study

This observational study consisted of two parts: a prevention study that determined the role of albumin infusion in the prophylaxis of HE and a treatment study that determined the role of albumin infusion in the treatment of overt HE.

### Ethics

This observational study was approved by the Medical Ethical Committee of the General Hospital of Northern Theater Command (formerly, the General Hospital of Shenyang Military Area). The ethical approval number is k (2018)18.

### Patient selection

We reviewed the medical records of cirrhotic patients who were consecutively admitted to the General Hospital of Northern Theater Command from January 2010 to June 2014. Cirrhosis was diagnosed based on clinical grounds involving laboratory tests, endoscopic findings, ultrasonographic findings, and liver histology, if available. Other eligibility criteria were as follows: 1) age and gender were not limited; 2) patients had no malignancy; 3) electronic medical records were complete; and 4) length of hospital stay was more than 24 hours. This was primarily because the patients who had been hospitalized for less than 24 hours were unable to receive effective and sufficient therapy. In the prevention study, the exclusion criteria were as follows: 1) patients with a diagnosis of overt HE at admission; and 2) patients who underwent transjugular intrahepatic portosystemic shunt (TIPS) or surgical shunt. In the treatment study, the exclusion criteria were as follows: 1) patients without overt HE at or after admission; 2) patients’ relatives declined further treatment or patients died immediately after a diagnosis of overt HE; and 3) patients who underwent TIPS or surgical shunt.

### Data collection

The primary data were collected as follows: age, gender, etiology of liver cirrhosis, ascites, acute upper gastrointestinal bleeding (AUGIB), infection, regular laboratory data, albumin infusion, albumin dosage, frozen plasma infusion, abdominal paracentesis, antibiotics, medications (i.e., lactulose, LOLA, compound amino acid, alanylglutamine, and arginine) for the prevention and treatment of overt HE, and in-hospital death. Child-Pugh [60] and MELD [61] scores were calculated. In the prevention study, we also recorded overt HE events during hospitalizations. In the treatment study, we also recorded the severity of overt HE at the time of diagnosis and the outcomes of overt HE after treatment during hospitalizations.

### Groups

In the prevention study, the patients were classified into two groups: 1) the albumin group in which patients received albumin infusion during the entire hospitalization or before a diagnosis of overt HE; and 2) the control group in which patients did not receive any albumin infusion during the entire hospitalization or before a diagnosis of overt HE. In the treatment study, the patients were classified into two groups: 1) the albumin group in which patients received albumin infusion after a diagnosis of overt HE; and 2) the control group in which patients did not receive any albumin infusion after a diagnosis of overt HE.

### Diagnosis and definitions

We reviewed the medical records to re-evaluate the diagnosis of overt HE according to the final report of 11^th^ World Congresses of Gastroenterology [62]. The grade of HE was assessed based on the West-Haven criteria [2]. Improvement of HE was defined as the grade of HE was decreased to a lower grade according to the West-Haven criteria.

### Outcomes

The outcomes of interest were the development and improvement of overt HE for the prevention and treatment studies, respectively. In-hospital mortality was also evaluated.

### Statistical analysis

A 1:1 PSM analysis was performed. Matching factors included age, sex, severity of liver dysfunction (Child-Pugh and MELD scores), ascites, AUGIB, infection, serum albumin at baseline, frozen plasma infusion, abdominal paracentesis, antibiotics, and drugs (lactulose, LOLA, compound amino acid, alanylglutamine, and arginine) for the prevention and treatment of overt HE. Continuous variables were reported as mean ± standard deviation and median (range) and the differences between albumin and control groups were compared by the non-parametric Mann-Whitney U test. Categorical variables were reported as frequency (percentage) and the differences between albumin and control groups were compared by the chi-square test. A two-tailed P<0.05 was considered statistically significant. All statistical analyses were performed with IBM SPSS 20.0 (IBM Crop) statistical package and Stata/SE 12.0 (Stata Corp, College Station, TX) software.

### Systematic review with meta-analysis

### Registration

The registration number of PROSPERO was CRD42018085605.

### Literature search

Three electronic databases (EMBASE, PubMed, and Cochrane Library) were searched from the earliest available publication until January 23, 2019. The following keywords were used: “albumin” and “hepatic encephalopathy”. No language restriction was applied. Only published data were considered. If some data was not available, we contacted with the corresponding authors to obtain the relevant data.

### Study selection

All potentially eligible studies should compare the outcomes of cirrhotic patients treated with and without albumin. The exclusion criteria were as follows: 1) duplicate articles; 2) reviews or meta-analysis; 3) case reports; 4) experimental or animal studies; 5) comments or letters; 6) guidelines or consensus; and 7) irrelevant topics.

### Data extraction

The following data were extracted: characteristics of included studies, baseline characteristics of patients, and outcome variables.

### Groups

In the prevention study, the albumin group should be that patients received albumin infusion; the control group should be that patients received standard medical treatment without additional albumin infusion. In the treatment study, the albumin group should be that patients received albumin infusion after a diagnosis of HE; the control group should be that patients did not receive albumin infusion after a diagnosis of HE.

### Outcomes

The primary outcomes were the development and improvement of overt HE for the prevention and treatment studies, respectively. The secondary outcomes were the changes of ammonia, IL-6, and TNF-α levels for the treatment studies.

### Study quality assessment

For RCTs, the Cochrane Risk of Bias tool was applied to assess the risk of bias from random sequence generation (selection bias), allocation concealment (selection bias), blinding of participants and personnel (performance bias), blinding of outcome assessment (detection bias), incomplete outcome data (attrition bias), selective reporting (reporting bias), and other sources. For non-RCTs, the Newcastle-Ottawa quality assessment scale was applied to assess the study quality, which evaluated a total of 8 indicators with the highest score of 9 points.

### Statistical analysis

The meta-analysis was performed using Review Manager (Version 5.2, Cochrane collaboration, The Nordic Cochrane Centre, Copenhagen) software and Stata/SE 12.0 (Stata Corp, College Station, TX) software. Dichotomous outcomes were expressed as OR with 95% CI; continuous outcomes were expressed as MD with 95% CI. Effect size estimates were analyzed using random-effect model. P<0.05 was considered statistically significant. Heterogeneity was assessed by the Cochrane Q test and the I^2^ statistics. P<0.1 or I^2^>50% was considered as a statistically significant heterogeneity. Publication bias was assessed by the Egger test. P<0.1 was considered as a statistically significant publication bias.

## Supplementary Material

Supplementary Figure

Supplementary Tables
