# Supplementary material for: Albumin infusion may decrease the incidence and severity of overt hepatic encephalopathy in liver cirrhosis
Source: Aging (Albany NY). 2019 Oct 8;11(19):8502–25. doi: 10.18632/aging.102335 (PMC6814610; doi:10.18632/aging.102335)
Supplement: Supplementary Figure [file aging-11-102335-s002.pdf]

SUPPLEMENTARY FIGURES

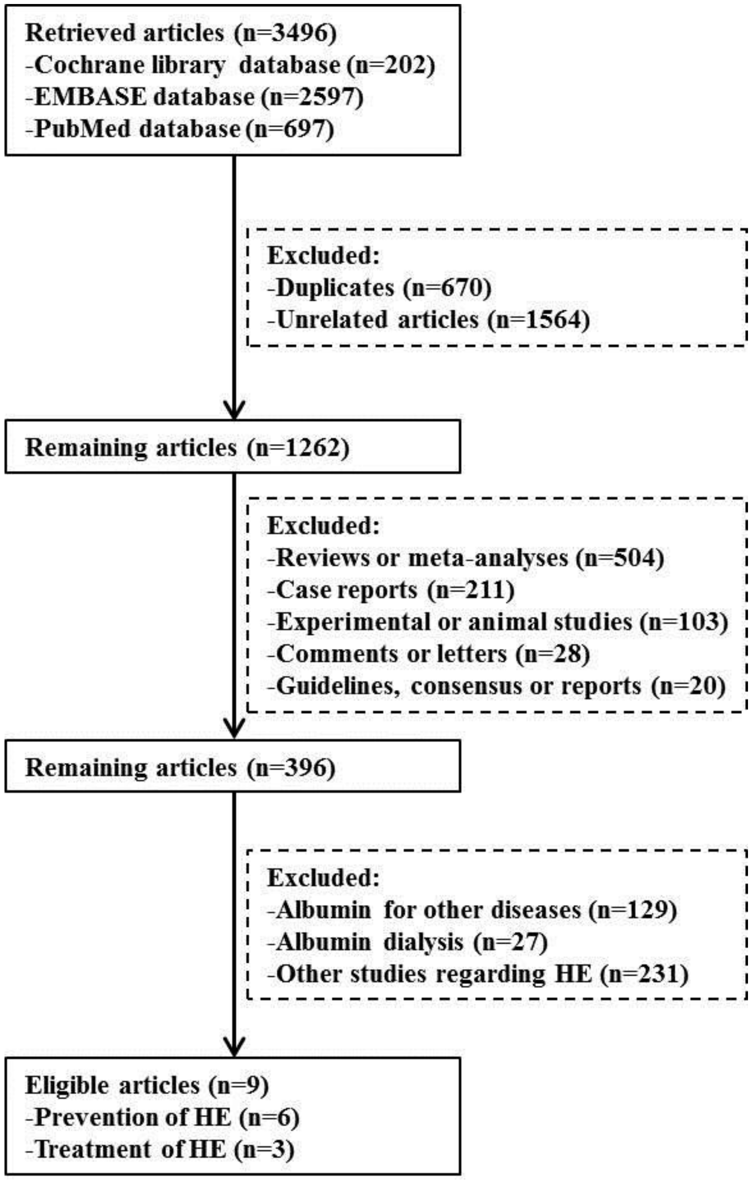

Supplementary Figure 1. Flow chart of study selection in our meta-analysis.

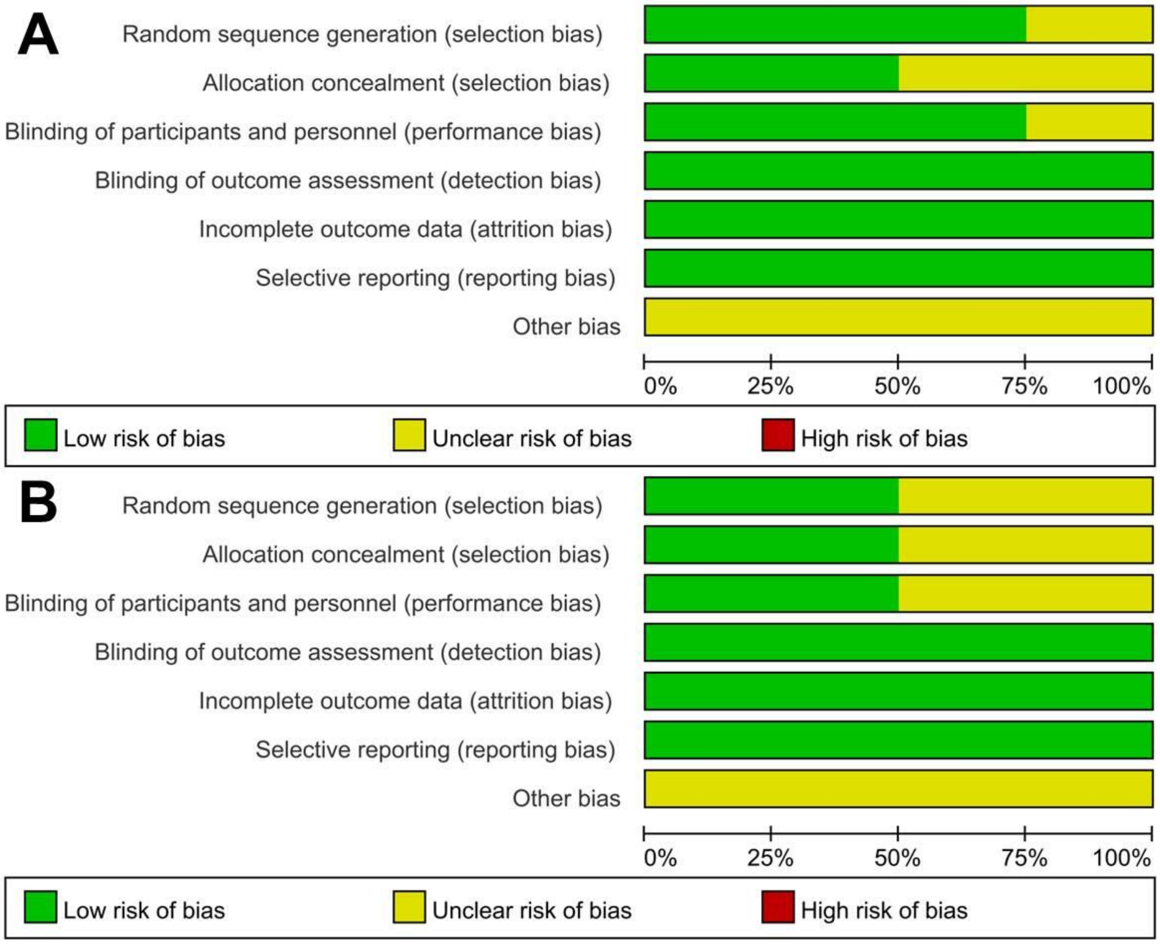

Supplementary Figure 2. Risk of bias of RCTs regarding the prevention (**A**) and treatment (**B**) of HE.

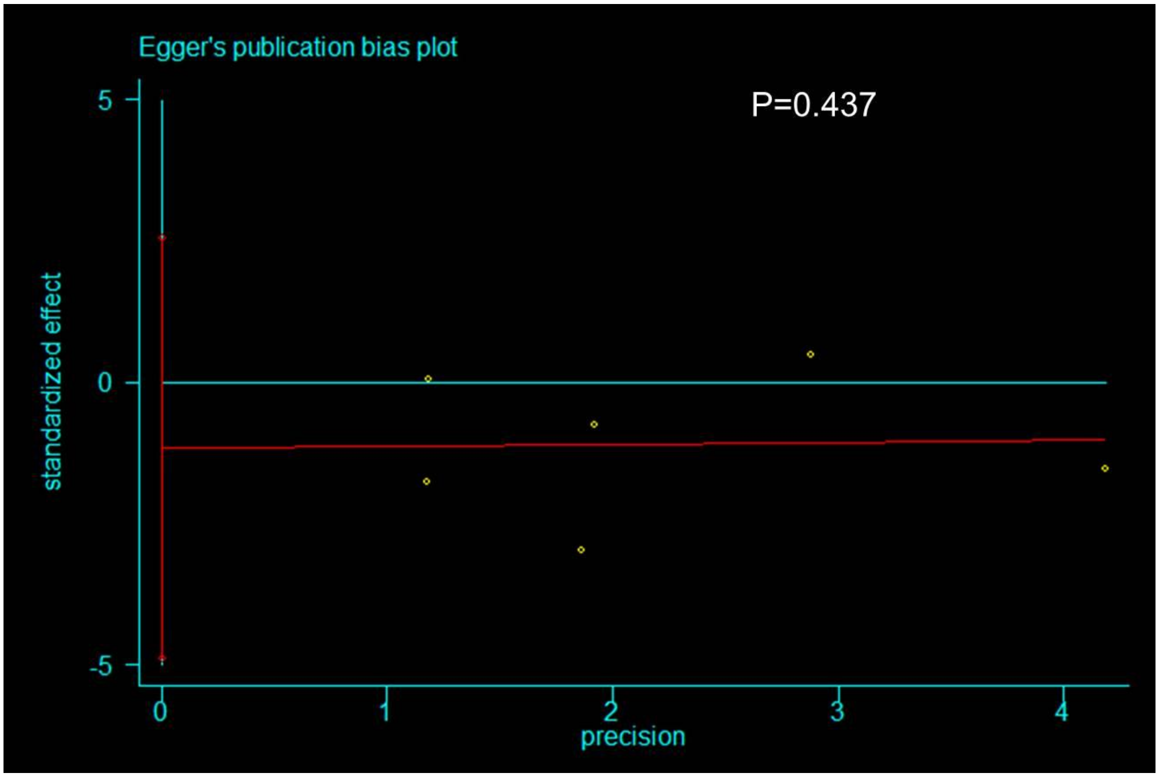

Supplementary Figure 3. Publication bias regarding the prevention of overt HE.

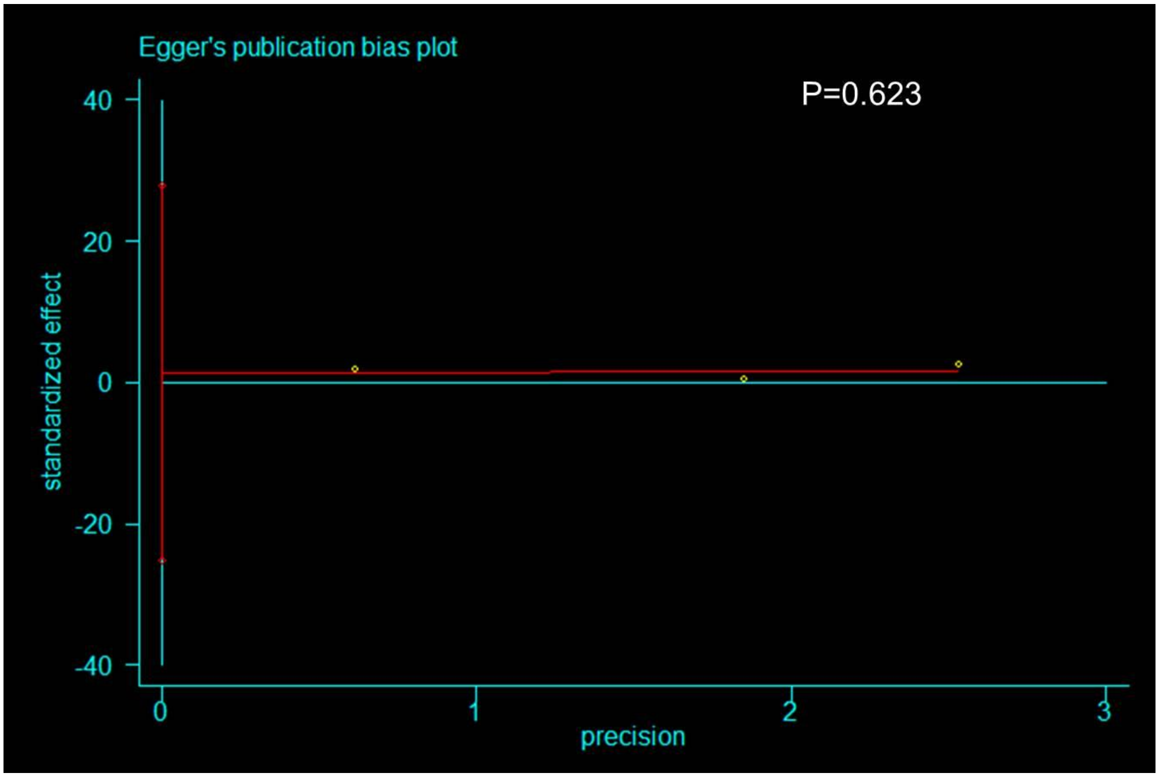

Supplementary Figure 4. Publication bias regarding the treatment of overt HE.

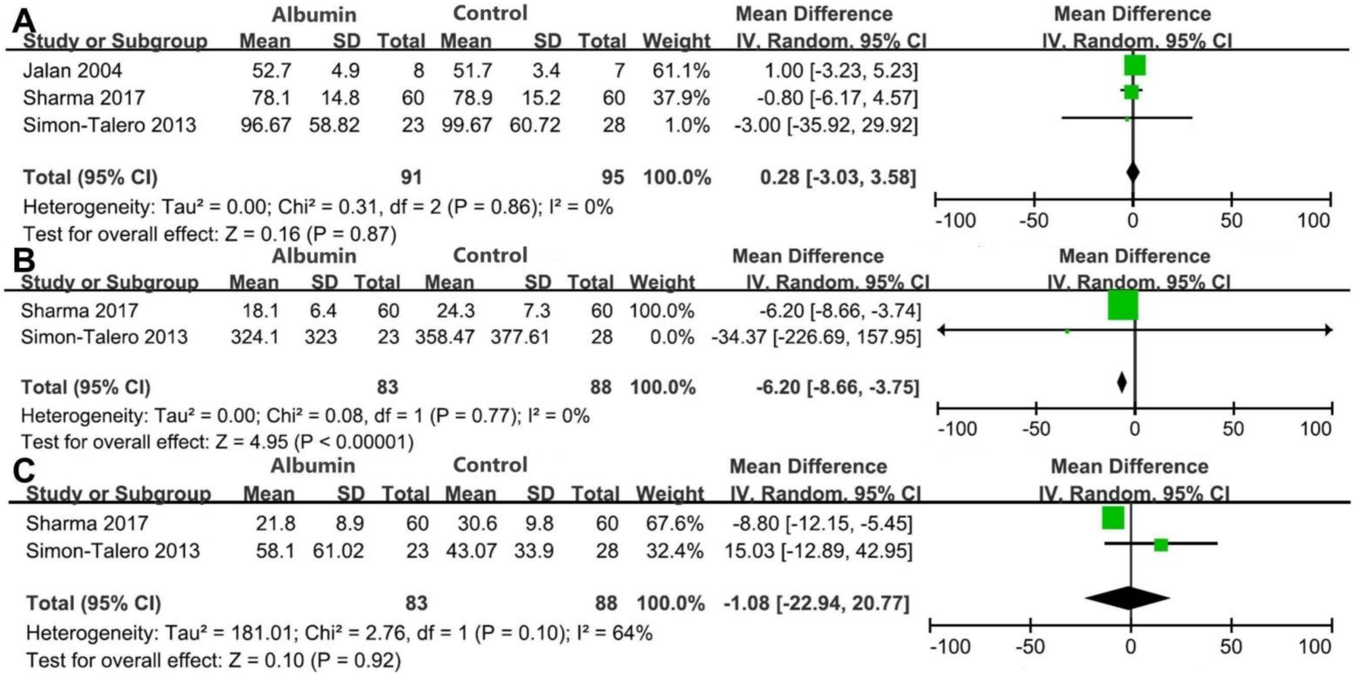

Supplementary Figure 5. Meta-analyses regarding ammonia (**A**), interleukin (IL)-6 (**B**), and tumor necrosis factor (TNF)-α (**C**) levels.

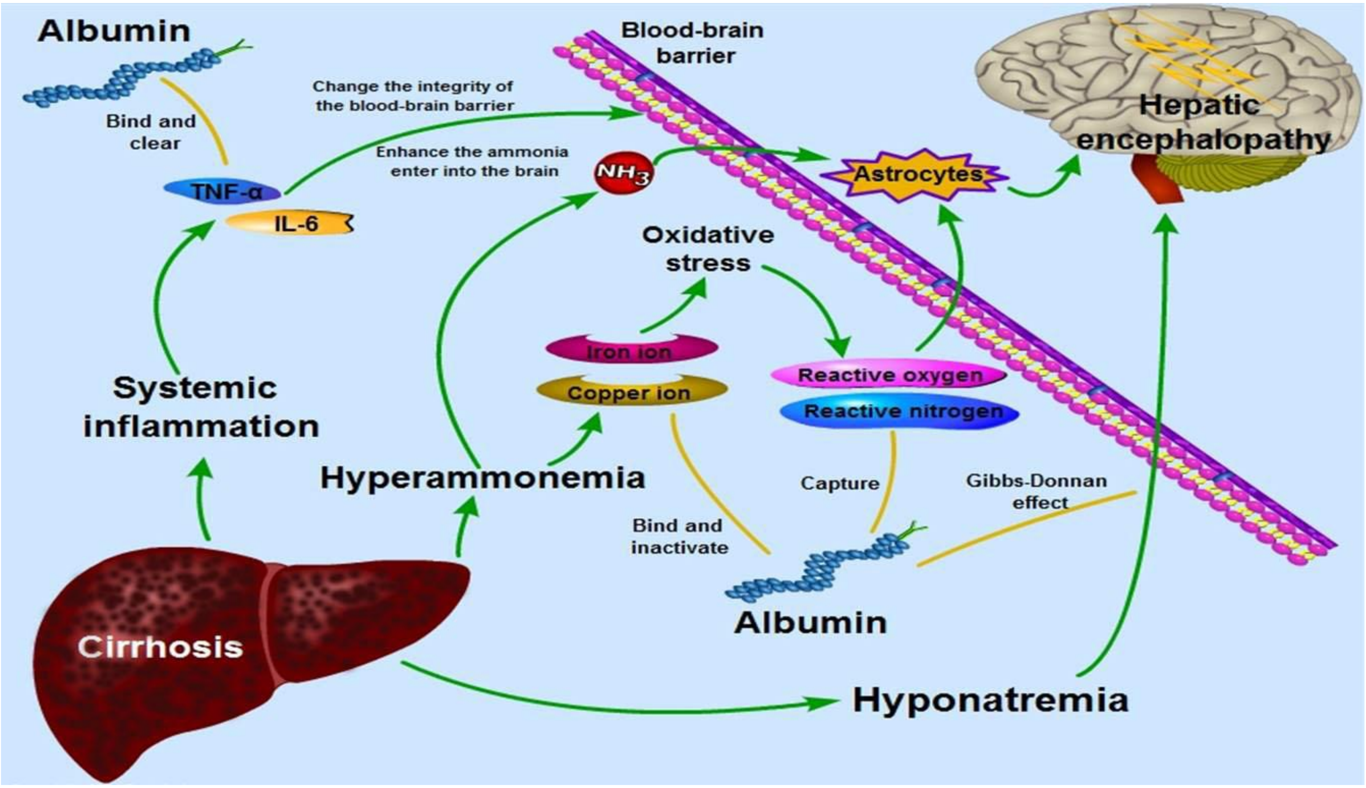

Supplementary Figure 6. Potential mechanisms of albumin for prevention and treatment of HE.
