# Supplementary material for: Albumin infusion may decrease the incidence and severity of overt hepatic encephalopathy in liver cirrhosis
Source: Aging (Albany NY). 2019 Oct 8;11(19):8502–25. doi: 10.18632/aging.102335 (PMC6814610; doi:10.18632/aging.102335)
Supplement: Supplementary Tables [file aging-11-102335-s001.pdf]

SUPPLEMENTARY TABLES

Supplementary Table 1. Inclusion and exclusion criteria of included studies.

First author (year)	Inclusion criteria	Exclusion criteria
Treatment study		
Jalan (2004)	1. HE of Grade 2 or higher (West Haven criteria).<br>2. HE was precipitated by dehydration (diuretic usage, oliguria, clinical evidence of dehydration and low central venous pressure).	1. Evidence of preadmission renal dysfunction.<br>2. Cardiac impairment or focal neurological abnormalities.<br>3. Any symptoms or signs of alcohol withdrawal.<br>4. Hepatic or extrahepatic malignancy.<br>5. Presence of other known precipitants of HE.<br>6. Administration of any specific therapy for HE, such as lactulose or bowel enemas, prior to enrolment.
Simon-Talero (2013)	1. Liver cirrhosis (diagnosed by clinical data or liver biopsy).<br>2. Development of an episode of HE that was initiated within 72h of inclusion into the study and persisted on grade ≥2 (West-Haven criteria).<br>3. Age between 18 and 85 years.	1. Terminal illness with a performance status ≥3 prior to HE.<br>2. Need for intensive support.<br>3. Comorbid psychiatric or neurological conditions that make the assessment of HE difficult.<br>4. Disorders requiring treatment with albumin.<br>5. Contraindication to albumin.<br>6. Active gastrointestinal bleeding in the previous 72h.<br>7. ACLF defined by an acute decompensation associated with bilirubin >5mg/dl.
Sharma (2017)	Patients with age 18-80 years with liver cirrhosis and overt HE.	1. Serum creatinine >1.5mg/dl.<br>2. Active alcohol intake <4 weeks prior to present episode.<br>3. Other metabolic encephalopathies.<br>4. HCC.<br>5. Degenerative central nervous or major psychiatric illness.<br>6. Significant co-morbidity.
Prevention study		
Planas (1990)	Cirrhosis with tense ascites.	1. Biochemical or echographic data suggesting HCC.<br>2. HE, gastrointestinal hemorrhage, or infection at entry.<br>3. Serum bilirubin count >10mg/dl.<br>4. Prothrombin time <40%.<br>5. Platelet count <40000/mm^3.<br>6. Serum creatinine concentration >3mg/dl.<br>7. Urinary sodium excretion rate >10mEq/day.
Riggio (2015)	All consecutive cirrhotic admitted to the author's Gastroenterology Unit to be submitted to TIPS were enrolled.	1. Past or present history of recurrent HE.<br>2. Serious cardiac or pulmonary dysfunction.<br>3. Diagnosis of HCC.<br>4. Sepsis.
Arora (2018)	NA	NA
Caraceni (2018)	1. Diagnosis of liver cirrhosis with uncomplicated ascites; ongoing diuretic treatment with an anti-aldosterone drug (at a dose ≥200mg/day and furosemide ≥25mg/day), stable for at least 4 days before enrolment.<br>2. Esophagogastroduodenoscopy done in the past 12 months, abdominal ultrasonography done in the past 30 days, and laboratory tests required by the protocol in the past 7 days.	1. Age <18 years.<br>2. Refractory ascites, recent complications of cirrhosis, TIPS, active HCC, liver transplantation, ongoing alcohol abuse, extrahepatic organ failure.<br>3. Albumin use for the treatment of ascites in the month preceding enrolment.

Sola (2018)	1. Age >18 years.<br>2. Cirrhosis defined by standard clinical, analytical and/or histological criteria.<br>3. Patients in the waiting list for liver transplantation.<br>4. Ascites.<br>5. Written informed consent.	1. Systolic arterial pressure ≥150mmHg and/or diastolic arterial pressure ≥90mmHg or drug therapy for arterial hypertension.<br>2. Treatment with psychotropic drugs or TIPS.<br>3. Treatment with antibiotics within the last 7 days prior to study inclusion except for norfloxacin or rifaximin as prophylaxis for SBP or recurrent HE, respectively.<br>4. Chronic heart or respiratory failure.<br>5. Listed for combined liver-kidney transplant.<br>6. Previous liver transplant.<br>7. HIV or HCV infection treated with antiviral agents.<br>8. Contraindications to receive midodrine.
Di Pascoli (2019)	1. Cirrhosis as diagnosed by liver biopsy or clinical, biochemical, ultrasound, and/or endoscopic findings.<br>2. Age >18 years.<br>3. Diagnosis of refractory ascites.	1. HCC or severe extrahepatic diseases.<br>2. Treatment with TIPS.

Abbreviations: HE: Hepatic Encephalopathy; TIPS: Transjugular Intrahepatic Portosystemic Shunt; ACLF: Acute-on-Chronic Liver Failure; HCC: Hepatocellular Carcinoma; h: Hours, NA: Not Available; HIV, Human Immunodeficiency Virus; HCV, Hepatitis C Virus; SBP: Spontaneous Bacterial Peritonitis.

Supplementary Table 2. Characteristics of patients.

First author (year)	Groups	Age (years)	Male/Female (n)	Etiology of cirrhosis (n)	Child-Pugh score	Child-Pugh (n)	MELD score
Treatment study							
Jalan (2004)	Albumin group	47.30±4.40	6/2	Alcohol: 7<br>Alcohol+HCV: 1	NA	B: 1<br>C: 7	NA
	Control group	50.10±6.10	4/3	Alcohol: 5<br>Alcohol+HCV: 2	NA	B: 1<br>C: 6	NA
Simon-Talero (2013)	Albumin group	63.70±11.30	19/7	Alcohol: 7<br>Virus: 9<br>Alcohol+Virus: 6	NA	NA	16.80±3.80
	Control group	66.30±9.70	23/7	Alcohol: 17<br>Virus: 10<br>Alcohol+Virus: 2	NA	NA	16.10±5.10
Sharma (2017)	Albumin group	42.50±8.70	49/11	Alcohol: 35<br>Virus: 17	9.70±1.90	B: 19<br>C: 41	26.40±5.80
	Control group	38.40±9.60	51/9	Alcohol: 32<br>Virus: 19	9.90±2.10	B: 17<br>C: 43	25.80±5.10
Prevention study							
Planas (1990)	Albumin group	59.00±1.50	25/18	Alcohol: 27	NA	NA	NA
	Control group	59.00±1.40	30/15	Alcohol: 32	NA	NA	NA
Riggio (2015)	Albumin group	57.70±10.00	17/6	Alcohol: 8<br>Virus: 9	NA	A: 7<br>B: 14<br>C: 2	11.50±3.30
	Control group	55.20±10.70	28/17	Alcohol: 18<br>Virus: 18	NA	A: 10<br>B: 25<br>C: 10	10.40±4.20
Arora (2018)	Albumin group	NA	NA	NA	NA	NA	NA
	Control group	NA	NA	NA	NA	NA	NA
Caraceni (2018)	Albumin group	61.00±11.40	146/72	Alcohol: 72<br>Virus: 63	8 (7-9)	A: 35<br>B: 141<br>C: 42	12 (10-15)
	Control group	61.40±10.90	150/63	Alcohol: 75<br>Virus: 69	8 (7-9)	A: 29<br>B: 141<br>C: 43	13 (10-16)
Sola (2018)	Albumin group	55.00±10.00	66/21	Alcohol: 34<br>Virus: 27	NA	NA	17.00±6.00
	Control group	54.00±11.00	71/15	Alcohol: 38<br>Virus: 27	NA	NA	16.00±6.20
Di Pascoli (2019)	Albumin group	64.20±10.40	31/14	NA	9.30±1.70	NA	14.90±5.00
	Control group	65.50±12.70	15/10	NA	9.50±1.60	NA	15.20±5.40

Abbreviations: HCV: Hepatitis C Virus; NA: Not Available.

Supplementary Table 3. Biochemical variables for treatment studies.

First author (year)	Groups		Albumin (g/dl)	Ammonia (µmol/L)	IL-6 (pg/ml)	Endotoxin (EU/ml)	TNF-α (pg/ml)
Jalan (2004)	Albumin group	Pre-treatment	2.71±0.32	98.00±7.30	NA	NA	NA
		Post-treatment	2.88±0.25	52.70±4.90	NA	NA	NA
	Control group	Pre-treatment	2.91±0.33	89.10±6.10	NA	NA	NA
		Post-treatment	2.74±0.45	51.70±3.40	NA	NA	NA
Simon-Talero (2013)	Albumin group	Pre-treatment	2.90±0.60	115.67±64.32	358.1±289.2	NA	71.27±90.83
		Post-treatment	NA	96.67±58.82	324.13±323	NA	58.10±61.02
	Control group	Pre-treatment	3.00±0.60	120.3±64.34	322.3±272.84	NA	47.93±46.47
		Post-treatment	NA	99.67±60.72	358.47±377.61	NA	43.07±33.90
Sharma (2017)	Albumin group	Pre-treatment	2.30±0.90	122.60±24.50	35.40±7.90	0.66±0.12	47.20±10.20
		Post-treatment	NA	78.10±14.80	18.10±6.40	0.25±0.08	21.80±8.90
	Control group	Pre-treatment	2.40±0.80	117.70±20.40	33.70±6.30	0.61±0.17	44.30±9.60
		Post-treatment	NA	78.90±15.20	24.30±7.30	0.38±0.07	30.60±9.80

Abbreviations: NA: Not Available, IL: Interleukin, TNF: Tumour Necrosis Factor.

Supplementary Table 4. Outcomes of albumin infusion for prevention of HE.

First author (year)	Groups	HE (n)	No HE (n)	Total (n)
Planas (1990)	Albumin group	3	40	43
	Control group	3	42	45
Riggio (2015)	Albumin group	9	14	23
	Control group	22	23	45
Arora (2018)	Albumin group	2	28	30
	Control group	7	22	29
Caraceni (2018)	Albumin group	39	179	218
	Control group	51	162	213
Sola (2018)	Albumin group	24	63	87
	Control group	21	65	86
Di Pascoli* (2019)	Albumin group	12	33	45
	Control group	16	9	25

Abbreviations: HE: Hepatic Encephalopathy.
Notes: *: Data was extracted from the main text.

Supplementary Table 5. Outcomes of albumin infusion for treatment of HE.

First author (year)	Groups	Improvement (n)	No Improvement (n)	Total (n)
Jalan (2004)	Albumin group	8	0	8
	Control group	3	4	7
Simon-Talero* (2013)	Albumin group	15	8	23
	Control group	16	12	28
Sharma (2017)	Albumin group	45	15	60
	Control group	32	28	60

Abbreviations: HE: Hepatic Encephalopathy,
Notes: *: Data was extracted from per-protocol analysis.
